# FFA2-, but not FFA3-agonists inhibit GSIS of human pseudoislets: a comparative study with mouse islets and rat INS-1E cells

**DOI:** 10.1038/s41598-020-73467-5

**Published:** 2020-10-05

**Authors:** Estela Lorza-Gil, Gabriele Kaiser, Elisabeth Rexen Ulven, Gabriele M. König, Felicia Gerst, Morgana Barroso Oquendo, Andreas L. Birkenfeld, Hans-Ulrich Häring, Evi Kostenis, Trond Ulven, Susanne Ullrich

**Affiliations:** 1 German Centre for Diabetes Research (DZD e.V.), Neuherberg, Germany; 2 Institute for Diabetes Research and Metabolic Diseases of the Helmholtz Center Munich at the University of Tübingen (IDM), 72076 Tübingen, Germany; 3 Internal Medicine IV, Endocrinology, Diabetology and Nephrology, University Hospital Tübingen, 72076 Tübingen, Germany; 4 Institute of Pharmaceutical Biology, University of Bonn, 53115 Bonn, Germany; 5 Department of Drug Design and Pharmacology, University of Copenhagen, 2100 Copenhagen, Denmark

**Keywords:** Lipid signalling, Diabetes, Target validation

## Abstract

The expression of short chain fatty acid receptors FFA2 and FFA3 in pancreatic islets raised interest in using them as drug targets for treating hyperglycemia in humans. This study aims to examine the efficacy of synthetic FFA2- and FFA3-ligands to modulate glucose-stimulated insulin secretion (GSIS) in human pseudoislets which display intact glucose responsiveness. The FFA2-agonists 4-CMTB and TUG-1375 inhibited GSIS, an effect reversed by the FFA2-antagonist CATPB. GSIS itself was not augmented by CATPB. The FFA3-agonists FHQC and 1-MCPC did not affect GSIS in human pseudoislets. For further drug evaluation we used mouse islets. The CATPB-sensitive inhibitory effect of 100 µM 4-CMTB on GSIS was recapitulated. The inhibition was partially sensitive to the G_i/o_-protein inhibitor pertussis toxin. A previously described FFA2-dependent increase of GSIS was observed with lower concentrations of 4-CMTB (10 and 30 µM). The stimulatory effect of 4-CMTB on secretion was prevented by the Gq-protein inhibitor FR900359. As in human pseudoislets, in mouse islets relative mRNA levels were *FFAR2* > *FFAR3* and FFA3-agonists did not affect GSIS. The FFA3-agonists, however, inhibited GSIS in a pertussis toxin-sensitive manner in INS-1E cells and this correlated with relative mRNA levels of *Ffar3* > > *Ffar2*. Thus, in humans, when FFA2-activation impedes GSIS, FFA2-antagonism may reduce glycemia.

## Introduction

The deorphanisation of the G-protein coupled receptors (GPCRs), GPR43 as FFA2 and GPR41 as FFA3, opens up a new pathway of interaction between the gut and the pancreatic islets^1–3^. Short chain fatty acids (SCFA) have been discovered to be physiological activators of FFA2 and FFA3^1^. SCFAs such as acetate, propionate and butyrate are mainly produced during fermentation of dietary fibres by the gut microbiome and are taken up into the blood stream^4,5^. In addition, acetate is the degradation product of ethanol metabolism in the liver as well as in other tissue. It has been suggested that alcohol dehydrogenases and aldehyde dehydrogenases are expressed in the human pancreas^6^. Like the long chain fatty acid receptor GPR40/FFA1, FFA2 and FFA3 are both expressed by the intestinal endocrine cells that produce the incretins GLP-1 and GIP^7–11^. The entero-endocrine-islet axis mediated by incretins has been studied in great depth^12^. Specific receptors on islet cells, i.e. GLP-1R and GIPR, transmit signals from the intestine to beta-cells, causing a potentiation of glucose-stimulated insulin secretion (GSIS)^13,14^. One of the beneficial effects of gut microbiome-derived SCFAs on metabolism is their stimulatory effect on GLP-1 and GIP secretion through FFA2 and FFA3 in entero-endocrine cells^7,10,15–17^. Direct effects of SCFAs on insulin secretion have been studied in isolated islets of wild type and receptor-deficient mice and insulin secreting cell lines. However, opposing effects on glucose homeostasis and insulin secretion have been observed^18–22^. These divergent results may be interpreted in various ways. Firstly, SCFAs are not only receptor agonists but also metabolites and can affect cellular metabolism depending on the experimental conditions^23^. Secondly, FFA2 couples to G_q_ and G_i/o_ proteins which are known to transmit stimulatory and inhibitory effects on insulin secretion, respectively, while FFA3 only couples to G_i/o_^24,25^. Thirdly, receptor expression on non-beta-cells within the islet may affect insulin secretion through paracrine effects^26,27^. Indeed, FFA2-agonists were recently reported to stimulate somatostatin secretion in mice^28^. While somatostatin is a potent inhibitor of insulin secretion, delta-cells are sparse and not uniformly distributed among or within the islets^29,30^.

The divergent effects could therefore depend on the expression levels of FFA2 and FFA3 in different islet cells. In view of the lack of specific antibodies for detection of endogenous FFA2 and FFA3 proteins, transcriptome analyses were used to estimate receptor’s expression. The mRNAs of FFA2 and FFA3 are discernible in isolated human islets using RT-qPCR^18,22^. Of note, in human single beta-cells the mRNA levels of FFA2 and FFA3 are barely detectable by RNAseq, suggesting that the expression of these receptors on beta-cells is low^31^. By comparison, the mRNA of FFA1 is highly abundant in beta- as well as in alpha- and gamma-cells^31^. It is worth bearing in mind that FFA2 and FFA3 are encoded in the same region of chromosome 19 (19q13) as FFA1 and that the same promoter region may regulate FFA1 and FFA3 transcription^1,24,32,33^. The different cellular levels of these mRNAs would then imply distinct posttranscriptional regulations.

In view of the contradictory effect of FFA2 and FFA3 on GSIS, a more detailed analysis, especially in human islets, is needed to understand the role of these receptors in glucose homeostasis in humans^34^. In previous studies, mainly SCFAs (acetate and propionate) were applied to isolated human islets^18–22^. In addition, FFA2-agonists which were used in two of these publications generated inconsistent results^19,22^. In the present study, we assessed the effects on GSIS of two FFA2-agonists (allosteric 4-CMTB and orthosteric TUG-1375), one FFA2-antagonist (CATPB), two FFA3-agonists (allosteric FHQC and orthosteric 1-MCPC) and of SCFAs (Table 1). In order to evaluate receptor expression and in view of the lack of specific and sensitive antibodies, relative mRNA levels of FFA2 and FFA3 were assessed in human islets, pseudoislets, mouse islets and INS-1E cells using semi-quantitative RT-PCR. Mouse islets and INS-1E cells were used for drug evaluation, in cases for unresponsiveness of human pseudoislets. We further discuss whether the small synthetic agonists or antagonists of FFA2 or FFA3 might be instrumental in improving insulin secretion under hyperglycaemic conditions in humans^35^.

**Table 1:** Properties of FFA2 and FFA3 synthetic agonists and antagonist.

Compound	Target	Chemical structure	Ligand affinity (pEC_50_)		References
			Human	Mouse	
4-CMTB	FFA2 allosteric agonist		hFFA2 (6.38) hFFA3 (NM)	mFFA2 (6.77) mFFA3 (NM)	^35–38^
TUG-1375	FFA2 orthosteric agonist		hFFA2 (7.10) hFFA3(NM)	mFFA2 (6.44) mFFA3 (NM)	^39^
CATPB	FFA2 antagonist		hFFA2 pIC_50_ 6.54	mFFA2 pIC_50_ NM	^36^
FHQC	FFA3 allosteric agonist		hFFA3 (5.65) hFFA2 (NM)	mFFA3 (5.20) mFFA2 (NM)	^40^
1-MCPC	FFA3 orthosteric agonist		hFFA2 (2.62) hFFA3 (3.88)	mFFA2 (2.22) mFFA3 (4.34)	^36, 41^

*NM* not measurable.

## Results

### In human pseudoislets FFA2-agonists inhibit GSIS

**Figure 1:**
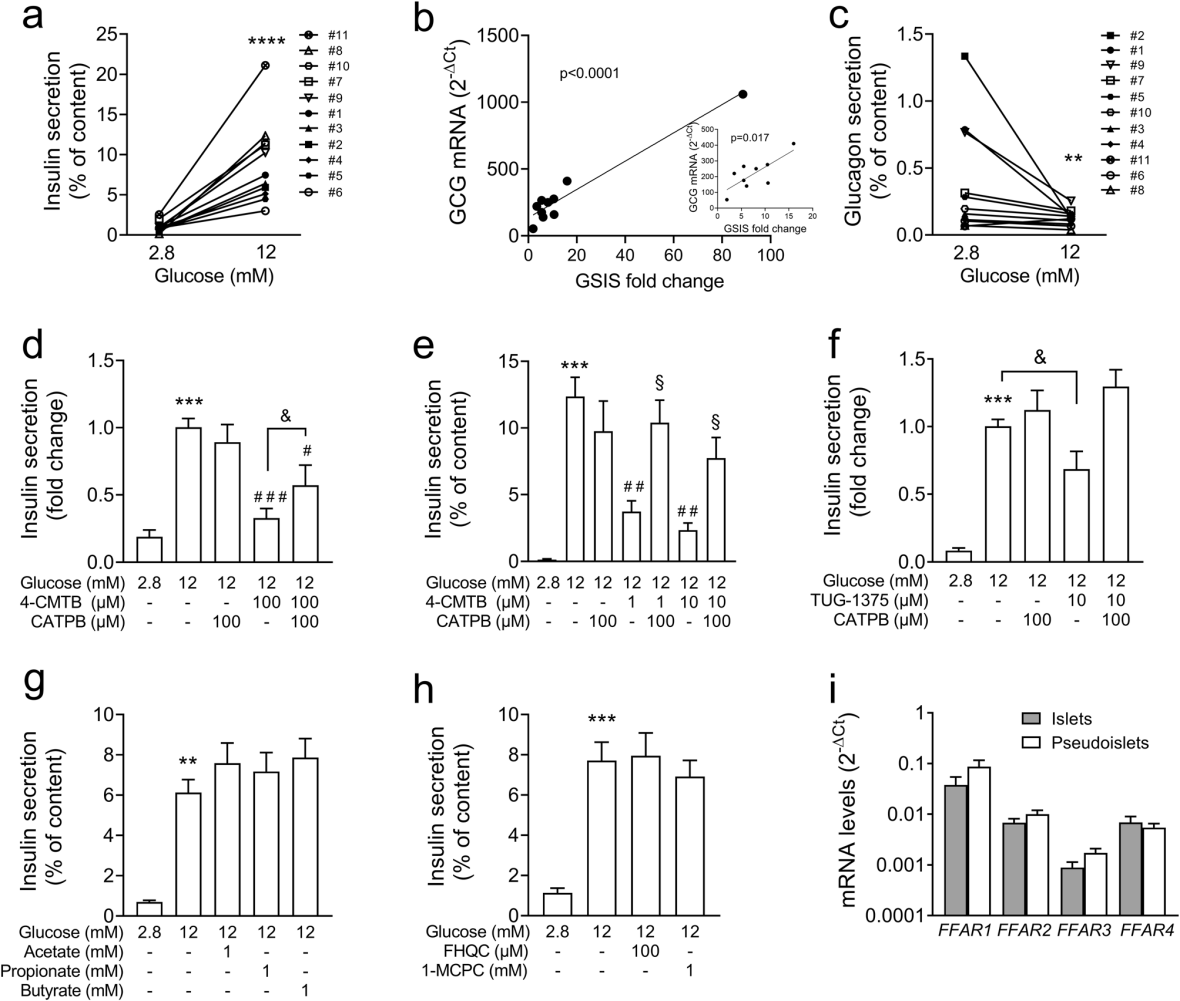
Effects of FFA2 and FFA3 ligands and SCFAs on GSIS in human pseudoislets. Human pseudoislets were prepared and incubated with test substances as indicated in each experiment and described under methods. (**a**,**d**–**h**) Insulin and (**c**) glucagon secretion are calculated as % of content and (**b**,**i**) relative mRNA levels are expressed as 2^−ΔCt^ (RPS13 was used as housekeeping gene). In insert in b the highest value is excluded. Results are presented as mean ± SEM of 4 replicates/conditions/donor of (**a**–**c**) n = 11, (**d**) n = 4, (**e**) n = 1, (**f**) n = 3, (**g**) n = 9, (**h**) n = 4, (**i**) n = 9 donors (see Supplementary Table S1). Significance **p < 0.01, ***p < 0.001 vs 2.8 mM glucose; ^#^p < 0.05, ^##^p < 0.01, ^###^p < 0.001 vs 12 mM glucose, ^§^p < 0.05 vs 12 mM glucose + 1 or 10 µM 4-CMTB, respectively, one-way ANOVA, followed by Tukey´s test. ^&^p < 0.05, unpaired Student’s t-test.

Adequate glucose responsiveness of insulin-secreting beta-cells is a prerequisite for functional testing. Previously, we described that reaggregation of isolated human islet cells into pseudoislets resulted in markedly improved GSIS^42^. The comparison of GSIS of isolated islets from human organ donors and of pseudoislets prepared thereof confirmed a better responsiveness of pseudoislets compared to islets (Fig. 1a, Table 2, Supplementary Fig. S1). In the pseudoislet preparations, insulin secretion at 12 mM glucose was ninefold higher than at 2.8 mM glucose (9.60 ± 0.93 (n = 41) and 1.09 ± 0.14 (n = 43) % of insulin content, respectively). The responsiveness was still variable, but did not correlate to the amount of stored insulin (Table 2). Interestingly, GSIS correlated positively to glucagon mRNA levels (Fig. 1b). In addition to an improved regulation of insulin secretion, glucagon secretion of pseudoislets was inhibited when raising glucose from 2.8 to 12 mM (Fig. 1c). Inhibition of glucagon secretion by raising glucose was not significant in isolated islets (Supplementary Fig. S1).

**Table 2:** Individual characteristics of human islets and the respective pseudoislet preparation.

Donor		SCFAs effect on GSIS	GSIS	GIGS	Ins content	Gcg content	mRNA levels (2^−∆Ct^)						
					ng	ng	INS	GCG	SST	FFAR1	FFAR2	FFAR3	FFAR4
#1	Islet		2.3	1.0	35.9 ± 1.36	0.92 ± 0.13	803.41	114.56	30.06	0.017	0.008	0.002	0.026
	PI	Stimulation	7.29	5.4	5.3 ± 0.36	0.45 ± 0.07	n.d	n.d	n.d	n.d	n.d	n.d	n.d
#2	Islet		2.4	2.4	14.4 ± 2.60	1.44 ± 0.40	639.14	99.04	17.03	0.132	0.007	0.000	0.003
	PI	Stimulation	10.6	22.5	7.8 ± 0.40	0.43 ± 0.09	617.37	159.79	32.90	0.257	0.009	0.000	0.002
#3	Islet		n.d	n.d	n.d	n.d	178.53	200.85	6.19	0.016	0.004	0.001	0.005
	PI	Stimulation	11.1	1.5	1.8 ± 0.15	0.67 ± 0.13	n.d	n.d	n.d	0.306	0.010	0.001	0.006
#4	Islet		1.96	1.5	7.3 ± 0.90	2.50 ± 0.34	349.71	53.08	11.08	0.011	0.003	0.001	0.002
	PI	No effect	5.5	1.4	6.0 ± 0.29	1.80 ± 0.38	284.05	265.03	10.78	0.043	0.007	0.002	0.003
#5	Islet		2.43	0.4	8.5 ± 1.29	1.73 ± 0.65	294.07	25.99	10.85	0.019	0.008	0.001	0.003
	PI	No effect	6.1	1.8	4.9 ± 0.26	0.91 ± 0.08	235.57	140.07	9.38	0.056	0.006	0.001	0.003
#6	Islet		n.d	n.d	n.d	n.d	n.d	n.d	n.d	n.d	n.d	n.d	n.d
	PI	No effect	3.5	1.6	4.1 ± 0.30	1.05 ± 0.07	278.00	220.00	21.60	0.067	0.020	0.003	0.016
#7	Islet		2.65	0.6	10.6 ± 1.70	0.72 ± 0.14	724.00	77.70	14.10	0.048	0.011	0.002	0.010
	PI	No effect	5.5	2.1	4.8 ± 0.30	0.60 ± 0.04	605.00	176.00	36.80	0.079	0.007	0.003	0.010
#8	Islet		n.d	n.d	n.d	n.d	n.d	n.d	n.d	n.d	n.d	n.d	n.d
	PI	Inhibition	88.6	1.8	7.8 ± 0.60	3.34 ± 0.90	1499.22	1060.11	58.08	0.044	0.133	0.003	0.136
#9	Islet		1.6	1.5	6.8 ± 2.40	1.00 ± 0.10	556.41	24.42	16.45	0.021	0.011	0.002	0.018
	PI	n.d	10.5	3.0	2.4 ± 0.22	0.33 ± 0.12	418.77	276.28	20.39	0.061	0.020	0.002	0.007
#10	Islet		2.3	2.4	14 ± 1.70	3.38 ± 1.05	211.00	25.60	7.01	0.018	0.003	0.001	0.007
	PI	n.d	16	1.4	2.3 ± 0.12	0.76 ± 0.07	159.00	410.00	6.50	0.081	0.013	0.003	0.007
#11	Islet		n.d	n.d	n.d	n.d	754.83	154.34	40.79	0.098	0.011	0.003	0.023
	PI	No effect	8.1	0.5	10.6 ± 0.60	1.8 ± 0.37	592.22	250.73	50.56	0.112	0.010	0.002	0.006

Data are expressed as mean ± SEM. PI, pseudoislet; SCFAs, short chain fatty acids; GSIS, glucose-stimulated insulin secretion (secretion at 2.8 mM was set to 1); GIGS glucose-inhibited glucagon secretion (secretion at 12 mM was set to 1).

Next, we tested the effects of FFA2- and FFA3-agonists on GSIS (Table 1). Both FFA2-agonists, 4-CMTB and TUG-1375, inhibited GSIS of pseudoislets (Fig. 1d–f). In 4 pseudoislet preparations 4-CMTB inhibited GSIS by 85% at the highest concentration tested (100 µM, Fig. 1d) while lower concentrations, 1 and 10 µM, had no effect (n = 4, not shown). Only in pseudoislets of donor#8, 4-CMTB efficiently inhibited GSIS at lower concentrations (1 and 10 µM, Fig. 1e). The FFA2-antagonist CATPB counteracted the inhibition induced by 4-CMTB confirming the FFA2-dependency. Neither FFA3-agonists FHQC and 1-MCPC nor SCFA significantly affected GSIS (Fig. 1g,h). Of note, the response to SCFAs (1 mM acetate, 1 mM propionate and 1 mM butyrate) was heterogeneous (Supplementary Fig. S2). In pseudoislets of 3 donors (donor #1–#3) all three SCFAs augmented GSIS by 55%, whereas an inhibitory effect on GSIS by acetate was observed in donor #8 only. In 5 preparations (donor #4–#7, #11) SCFAs did not affect GSIS. Thus, heterogeneous effects of acetate are not a consequence of inconsistent experimental conditions between laboratories, but reflect differences between patients.

In accordance with the FFA2-effects on GSIS in pseudoislets, the relative mRNA levels of *FFAR2* were always higher than those of *FFAR3,* especially in donor #8 that showed a pronounced acetate- and FFA2-agonist mediated inhibition of GSIS (Fig. 1i, Table 2). Of note, the mRNA levels of *FFAR1, FFAR2, FFAR3* and *FFAR4* as well as of *INS* (insulin), *GCG* (Glucagon) and *SST* (somatostatin) were comparable between pseudoislets and isolated islets of the same human donors (Fig. 1i, Table 2). These results suggest that FFA2- and FFA3-agonists are unsuitable for the treatment of insufficient insulin secretion in humans. FFA2-antagonists, in contrast, may augment GSIS, but only under conditions of FFA2-dependent inhibition of insulin secretion.

### In mouse islets FFA2-agonist 4-CMTB exerted a dual concentration-dependent effect on GSIS through distinct pathways

**Figure 2:**
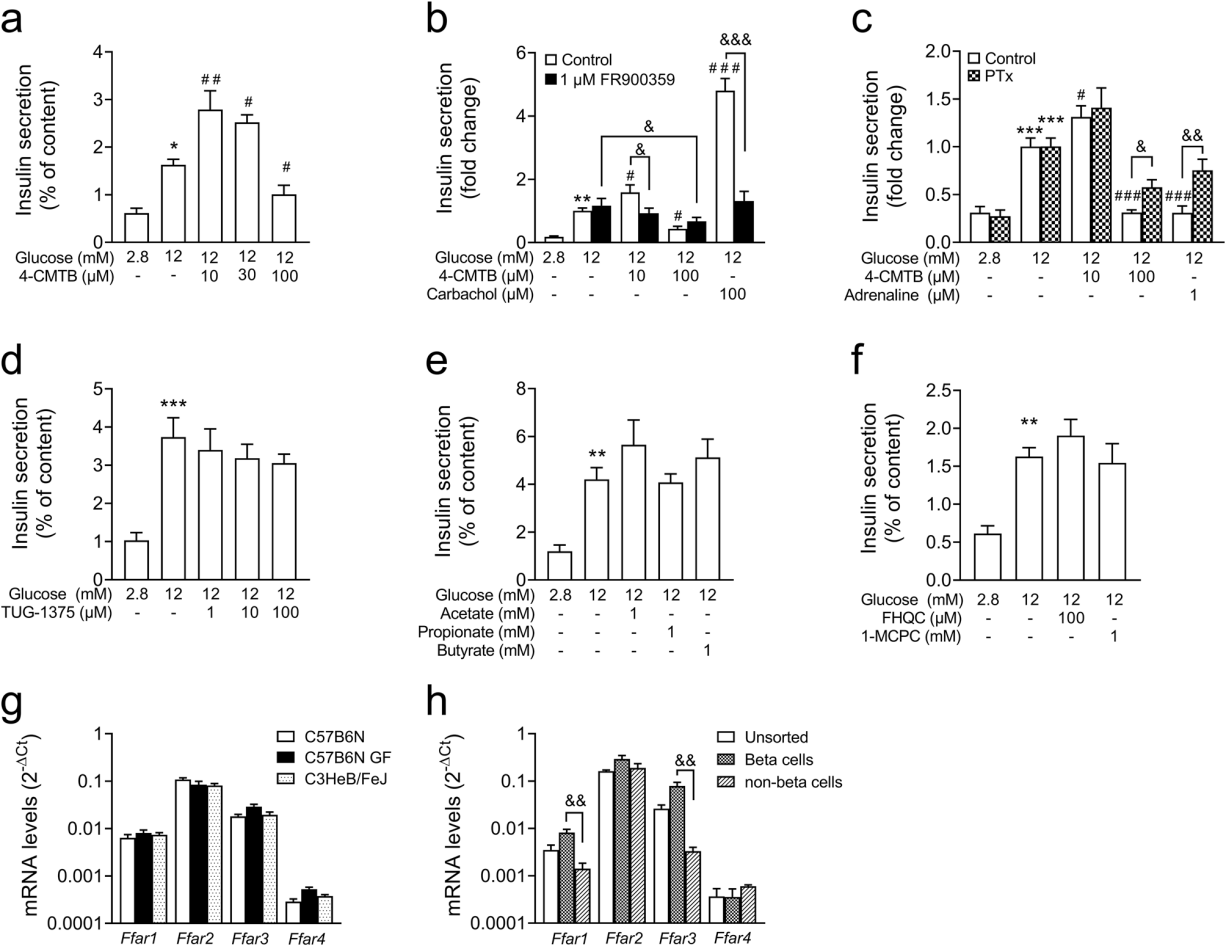
Effects of FFA2 and FFA3 ligands and SCFAs on GSIS in mouse islets. (**a**–**f**) Mouse islets were isolated, overnight cultured and incubated with test substances as indicated in each experiment and described under methods. (**b**) FR900359 (G_q/11_ inhibitor) was added into the preincubation and incubation solution. (**c**) Pertussis toxin (PTx) pretreatment (100 ng/ml) was performed in culture 24 h before the experiments. (**g**) RNA was prepared from freshly isolated mouse islets and (**h**) from freshly isolated islet cells as described under methods. (**a**–**f**) Insulin secretion is expressed as % of content or relative to 12 mM glucose, (**g**,**h**) relative mRNA levels are expressed as 2^−ΔCt^ (RPS13 was used as housekeeping gene). Results are presented as mean ± SEM of n = 3 C57BL/6N mouse islet preparations with 4 replicates/condition for insulin secretion; n = 3–7 of C57BL/6N for mRNA analysis. C57B6N: C57BL/6N; GF: Germ-free mice. Significance *p < 0.05, **p < 0.01, ***p < 0.001 vs 2.8 mM glucose, ^#^p < 0.05, ^##^p < 0.01, ^###^p < 0.001 vs 12 mM glucose, one-way ANOVA, followed by Tukey’s test. ^&^p < 0.05, ^&&^p < 0.01, ^&&&^p < 0.001 unpaired Student’s T-test.

In view of the unresponsiveness of the human preparations to FFA3-agonists and the absence of stimulatory effects of FFA2-agonists on GSIS, we evaluated the ligands in established rodent cell models, i.e. isolated mouse islets and INS-1E cells. This is possible since the FFA2- and FFA3-agonists have similar affinities and selectivity to mouse as to human receptors (Table 1). In contrast to the consistent inhibitory effect of 4-CMTB in human pseudoislets, in mouse islets the FFA2-agonist 4-CMTB displayed a concentration-dependent dual effect on GSIS (Fig. 2a). At low concentrations, 10 and 30 µM, 4-CMTB stimulated GSIS, while addition of 100 µM inhibited secretion. To examine whether the dual effect of 4-CMTB is due the activation of different G-protein regulated signalling pathways, the effects of 4-CMTB on GSIS was analysed in the presence specific inhibition of G_q/11_-proteins with FR900359^43^ and of G_i/o_-proteins with pertussis toxin^44^. Preincubation of mouse islets with the G_q_-inhibitor FR900359 counteracted the stimulation of insulin secretion induced by 10 µM 4-CMTB (Fig. 2b). Of note, FR900359 abolished muscarinic acetylcholine receptor M3-dependent augmentation of GSIS by carbachol. In contrast, the inhibition of GSIS by 100 µM 4-CMTB was still significant in the presence of FR900359, but partly reversed by pertussis toxin (PTx) pretreatment (Fig. 2c). As control, adrenaline, a potent physiological inhibitor of GSIS, was used, which activates alpha2-adrenergic receptors linked to a PTx-sensitive G-protein^45^. Pertussis toxin treatment was efficient, since adrenaline-mediated inhibition of GSIS was no longer significant in PTx-treated mouse islets (Fig. 2c). The orthosteric FFA2-agonist, TUG-1375 which mimics the interaction of SCFAs with the receptor, did not influence GSIS at 1, 10 and 100 µM (Fig. 2d). Neither SCFAs nor FFA3-agonists affected GSIS (Fig. 2e,f). These results confirm that in mouse islets FFA2-agonist can activate a stimulatory but also an inhibitory pathway.

As in human islets, in mouse islets relative mRNA levels of *Ffar2* were higher than of *Ffar3* (Fig. 2g). The order of relative mRNA abundance was *Ffar2* > *Ffar3* > *Ffar1* > > *Ffar4*. The *Ffar1-4* mRNA levels of isolated islets of different mouse strains (C3HeB/FeJ and C57BL/6N) were comparable and did not change significantly when the mice were held under germ-free conditions (Fig. 2g). Using FACS-sorted GFP-labelled insulin-producing cells of C57BL/6N RIP-Cre mT/mG mice, *Ffar2* mRNA levels were discernible in the beta- and non-beta-cell fractions, while *Ffar3* mRNA was enriched in beta-cells (Fig. 2h).

These results suggest that the regulation of insulin secretion is comparable between human pseudoislets and mouse islets in regard to the inhibitory effect of high concentrations of FFA2-agonists and unresponsiveness to FFA3-agonists. A stimulatory effect of 4-CMTB on GSIS is only observed in mouse islets.

### In rat INS-1E cells SCFAs and FFA3 agonists inhibit GSIS

**Figure 3:**
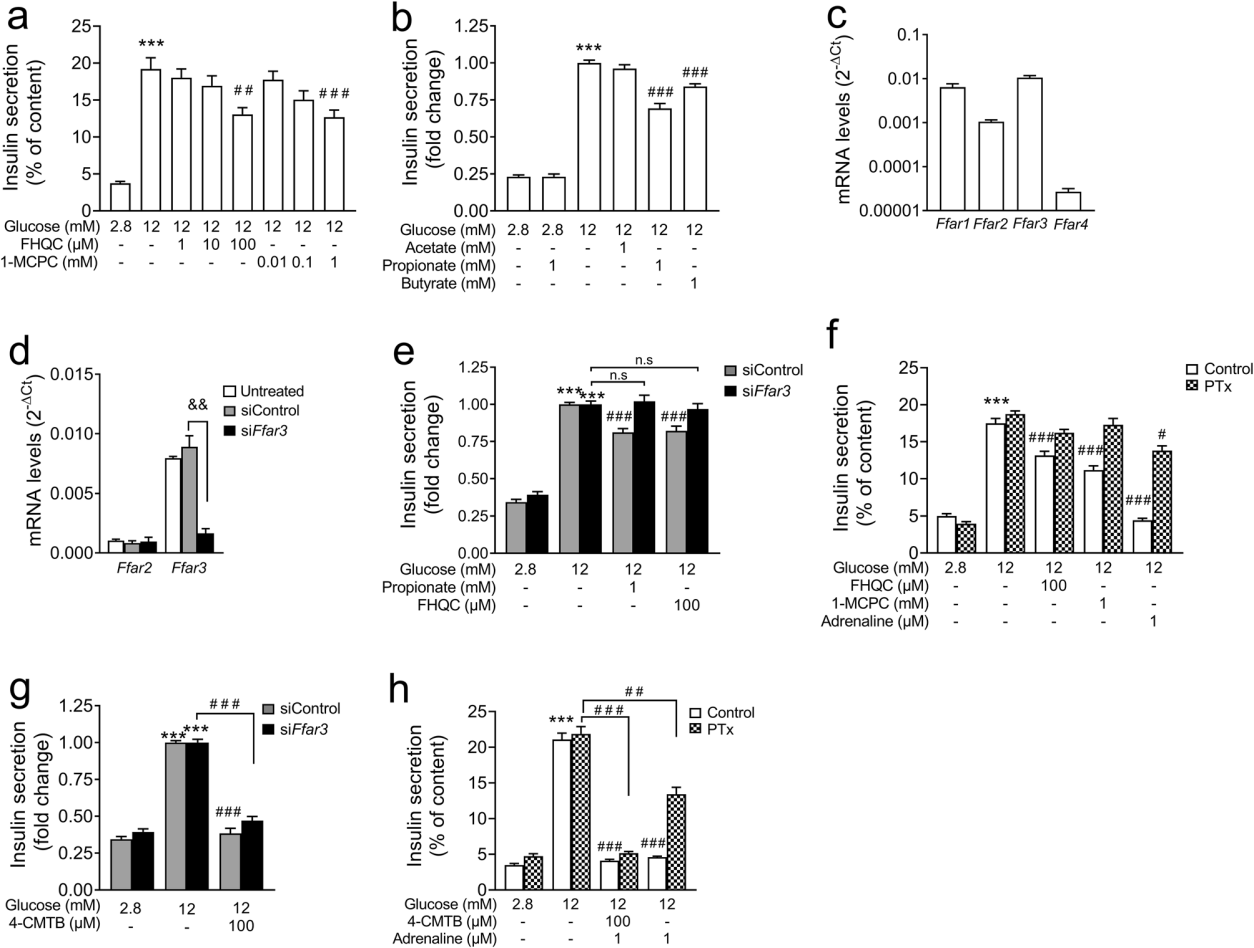
Effects of FFA2 and FFA3 ligands and SCFAs on GSIS in INS-1E cells. (**a**–**h**) INS-1E cells were cultured and incubated with test substances as indicated and described under methods. (**d**,**e**,**g**) INS-1E cells were untreated (white bars), treated with non-targeting siRNA (grey bars) and treated with *Ffar3* siRNA (black bars) as described under methods. (**f**,**h**) INS-1E cells were cultured in the presence of pertussis toxin (PTx;100 ng/ml) for 24 h before the experiments. (**a**,**f**,**h**) Insulin secretion is expressed as % of content or (**b**,**e**,**g**) relative to secretion at 12 mM glucose. (**c**,**d**) mRNA levels are expressed as (2^−ΔCt^). RPS13 was used as housekeeping gene. Results are presented as mean ± SEM of n = 3–4 independent experiments. Significance ***p < 0.001 vs the respective control at 2.8 mM glucose; ^#^p < 0.05, ^##^p < 0.01, ^###^p < 0.001 vs the respective stimulation at 12 mM glucose, one-way ANOVA followed by Tukey’s test; *n.s* not significant.

Since FFA3-agonists had no effect on GSIS in human pseudoislets and mouse islets, we tested FHQC and 1-MCPC on GSIS in INS-E cells, a rat insulin-secreting cell line. At the highest concentration tested FHQC and 1-MCPC significantly reduced GSIS (Fig. 3a). A comparable inhibitory effect on GSIS was exerted by 1 mM propionate and 1 mM butyrate but not by acetate (Fig. 3b). In INS-1E cells, the relative mRNA levels of *Ffar3* were remarkably high, those of *Ffar2* were low (Fig. 3c). This expression pattern could explain the inhibitory effect of propionate and butyrate on GSIS in INS-1E cells, since the potency of FFA3 activation declines from butyrate = propionate > > acetate^1,36^. Since no specific FFA3-antagonist was available, the expression of FFA3 was reduced by transfecting INS-1E cells with siRNA against FFA3 (Fig. 3d). The efficient downregulation of FFA3 (by 85%) abrogated the inhibitory effect of FHQC and propionate on secretion without affecting GSIS (Fig. 3e).

Next, we examined whether the FFA3-dependent inhibition of GSIS was transmitted via a PTx sensitive G_i/o_ protein as suggested previously^18^. After PTx pretreatment of the cells, both agonists, FHQC and 1-MCPC, were unable to reduce GSIS (Fig. 3f). PTx also abrogated the inhibitory effect of adrenaline. In comparison to the pronounced inhibitory effect of adrenaline on secretion, the FFA3-agonist attenuated secretion by 40%.

Although *Ffar2* mRNA levels were low, we tested the FFA2-agonist 4-CMTB in INS-1E cells. 4-CMTB at 100 µM inhibited GSIS similar to the inhibition observed in mouse islets and human pseudoislets (Fig. 3g). 4-CMTB still efficiently inhibited GSIS in the presence of siRNA against FFA3 or after PTx pretreatment of the cells (Fig. 3g,h).

In summary, FFA3-agonists FHQC and 1-MCPC inhibit GSIS through a PTx-sensitive G-protein in INS-1E cells. The ineffectiveness of the FFA3-agonists and the low abundance of *FFAR3* mRNA especially in human pseudoislets suggest that FFA3 is not functionally expressed in human beta-cells. FFA2-agonists consistently inhibited insulin secretion at high concentration (100 µM) in all cell systems tested.

## Discussion

This study revealed FFA2-antagonist by preventing FFA2-mediated inhibition of GSIS as putative therapeutic targets for the treatment of hyperglycaemia. The results further suggest that FFA3 is not functionally expressed in human islets since FFA3 agonists did not affect GSIS and mRNA levels of *FFAR3* were very low, i.e. at the detection limit.

In human pseudoislets as well as in mouse islets and in INS-1E cells, the FFA2-agonist 4-CMTB invariably inhibited GSIS at 100 µM. In contrast to mouse islets, where 10 µM 4-CMTB augmented GSIS, in human pseudoislets, 4-CMTB at lower concentrations inhibited GSIS or had no effect. The concentration-dependent dual effect elicited by 4-CMTB in mouse islets, was observed in a Min6 cell preparation with another FFA2-agonist and might be, therefore, mouse specific^46^. Another, structural related FFA2-agonist stimulated GSIS in human islets at 1 µM concentration^22^. Using a higher number of donor preparations (n=11, Fig. 1) compared with that ones used  by McNelis (n=3)^22^, we observed highly variable effects. Therefore, this stimulatory effect might be donor specific.

The inhibition of GSIS by 4-CMTB was efficiently reversed by the FFA2-antagonist CATPB confirming that the effect of 4-CMTB was transmitted through FFA2. Furthermore, in INS-1E cells siRNA against Ffar3 did not affect the inhibition by 4-CMTB. Since the antagonist had no effect on GSIS, a favourable effect on insulin secretion is expected only under FFA2-stimulation. This conclusion endorses the proposal made by Stefan Offermann that FFA2-antagonists may be useful for the treatment of hyperglycaemic episodes by improving insulin secretion but our results suggest utility limitations as the antagonist only counteracted FFA2-mediated inhibition of GSIS^18^.

Physiological stimuli of FFA2 and FFA3 so far identified are SCFAs, acetate, propionate and butyrate. Using static incubations, we observed a heterogeneous response of human pseudoislets to SCFAs. A significant inhibition of GSIS comparable to the 4-CMTB-induced inhibition was observed in one out of 9 preparations. This inhibition by acetate and low concentration of 4-CMTB correlated with 10-times higher relative mRNA levels of FFA2 compared to the other donors (2^−ΔCt^ of 0.133 vs < 0.02, respectively; Table 2). Due to restricted information available from organ donors we are unable to speculate about the reason of this heterogeneous expression of FFA2 and response to SCFAs. Acetate, the main SCFA in blood derives mainly from the gut microbiome or from alcohol consumption. That the gut microbiome does not impact on FFA2 and FFA3 expression in islets is corroborated by the finding that FFA2 and FFA3 mRNA levels were not affected by the maintenance of mice under germ-free conditions. Alcohol consumption increases blood acetate levels above 1 mM and associates with decreased fasting and 2 h-postload insulin levels compared to non-drinker^47,48^. In contrast to acetate, plasma concentrations of propionate and butyrate remain at low micromolar range, concentrations which do not activate the receptors.

Our results confirm previous observations that both stimulatory and inhibitory effects can be triggered by SCFAs, and suggest that this heterogeneity is not just a result of different experimental settings^49^. In three previous studies using static incubations of human islets, acetate either inhibited^18^ or did not alter GSIS^19,22^. In other studies using perifused human islets, acetate and propionate potentiated GSIS^20,21^. We found in static incubations of pseudoislets stimulation, inhibition or no effect of SCFAs on GSIS, even though glucose stimulated insulin secretion to the same extent and the *FFAR2* and *FFAR3* mRNA levels were similar in responsive and unresponsive pseudoislets.

Another explanation for the heterogeneous effect of SCFAs and FFA2 agonists on GSIS in human pseudoislets could be a heterogeneous composition of beta:alpha:delta-cells. Somatostatin (from delta-cells) is a potent inhibitor not only of insulin but also of glucagon secretion in humans and rodents^30^. On average, less than 10% of the endocrine islet cells are delta-cells. A recent publication convincingly demonstrated that FFA2-agonists stimulate somatostatin secretion in mice^28^. FFA2 may be expressed in delta-cells, as suggested by the expression pattern of FACS-separated mouse beta-cells and non-beta-cells (Fig. 2h). A variable number of delta-cells may affect the efficiency of the paracrine inhibition of GSIS by somatostatin. In the pseudoislet preparation of donor #8 FFA2-agonists and acetate potently inhibit GSIS and the mRNA levels of somatostatin and FFA2 were higher compared to pseudoislets of the other donors. However, somatostatin acts via PTx-sensitive pathways. In mouse islets, the inhibitory effect of high concentrations of 4-CMTB was only partially reversed by PTx. This indicates both, an activation of a receptor which links to PTx-sensitive G_i/o_ proteins but also the involvement of a PTx-insensitive pathway as has been observed previously^19^.

We decided to use pseudoislets, since they display a much better GSIS than the isolated islets from the same human donor^42^. The mRNA levels of insulin, somatostatin and of the fatty acid receptors *FFAR1-4*, were comparable between islets and pseudoislets, suggesting that expression of hormones and receptors is not significantly different between islets and pseudoislets (Table 2)^42^. Interestingly, glucagon mRNA levels were higher in pseudoislets compared to islets yielding in a positive correlation between glucagon mRNA levels and insulin secretion. A higher glucose responsiveness has been found in glucagon rich, dorsal islets compared to glucagon poor, ventral islets isolated from the same rat suggesting that islet glucagon contributes to higher glucose responsiveness^50^. Further studies will help to understand the mechanism underlying the increased production of glucagon in pseudoislets.

The specificity of FFA2-agonists, the FFA2-antagonist^37–39,51,52^ and FFA3 agonists, FHQC and 1-MCPC^53,54^ have been evaluated in expression systems and, at the concentrations used, they activate the respective murine and human receptors (Table 1). However, the FFA3-agonists had no significant effect on GSIS in human pseudoislets and mouse islets. Only in INS-1E cells a significant inhibitory effect on GSIS was observed and correlated with higher mRNA levels of *Ffar3* compared to *Ffar2*. Thus, the absence, or very low expression of FFA2 in INS-1E cells could uncover a FFA3-dependent inhibition of GSIS. The assumption that the relative ratio of FFA2/FFA3 expression in beta-cells determines stimulation or inhibition may explain the results obtained in transgenic (tg) mice overexpressing FFA3^55^. In the FFA3 tg mice, i.e. 40-fold higher expression of FFA3 over FFA2 in beta-cells, plasma glucose excursions during glucose stimulation (oGTT) were increased (due to FFA3-mediated inhibition of GSIS), whereas the absence of FFA3 slightly attenuated the elevation of plasma glucose during oGTT (due to FFA2-mediated stimulation of GSIS). Accordingly, the deletion of FFA2 was not sufficient to overcome the inhibitory effect of acetate on GSIS in isolated mouse islets^18^. Only a deletion of both FFA2 and FFA3 overcame the inhibitory effect of acetate on insulin secretion. The results in our sorted mouse islet cells suggest that FFA3 is enriched in the beta-cell fraction, while FFA2 is expressed on beta- and non-beta-cells. The reason for the very different *Ffar2* mRNA levels (more than 2 orders of magnitude) of mouse islets and rat INS-1E cells remains elusive, but expression of FFA2 in non-beta-cell population could contribute to the high islet mRNA levels.

In conclusion, although SCFAs have direct effects on insulin secretion in human islets, these effects are highly heterogeneous among individuals. While SCFAs indirectly augment GSIS by increasing incretin secretion, they may inhibit insulin secretion and subdue the incretin effects via direct effects within the islets^56^. Further experimental evidence is needed to determine whether the beneficial metabolic effects of fibre ingestion include SCFA-effects on beta-cell’s differentiation, survival and protection against stress factors^57,58^. This study suggests that FFA2, but not FFA3, is functionally expressed in human islets and that FFA2-antagonists may exert beneficial effects on hyperglycaemic episodes by counteracting FFA2-dependent inhibition of GSIS.

## Methods

### Human islet and pseudoislet preparations

Human pancreatic islets from organ donors were provided by the European Center for Islet Transplantation (ECIT, JDRF award 31-2008-416 for basic research programme) or purchased from Tebu-Bio (Offenbach, Germany). Donors gave informed consent for the use of their islets preparations in scientific research. The use and the procedures and protocols involved in handling of human islets were approved by the Ethics Commission of the Medical Faculty of the Eberhard Karls University and the University Hospital Tübingen (098/2017BO1). All experiments involving human material were performed in accordance with the above mentioned approvals, guidelines and regulations. The characteristics of human pancreatic donors are provided in Supplementary Table S1. The islets were cultured overnight in CMRL1066, containing 5 mM glucose, 10% (v/v) FCS (Serva, Heidelberg, Germany), 10 mM HEPES, 2 mM l-glutamine, and 1% penicillin/streptomycin at 37 °C in a 5% CO_2_-humidified atmosphere. The pseudoislets were prepared after dissociation of islets into single cells with 0.25% Trypsin–EDTA in PBS at 37 °C for 5 min as already described in detail^42^. In brief, 2000 cells were reaggregated in hanging drops of 20 µl medium on the top of a petri dish. After 3d of culture, the reaggregated pseudoislets were harvested and placed into 24-well plates with one pseudoislet in 0.5 ml medium/well and cultured for further 2d.

### Mouse islets and FACS isolated beta-cells

Islets from adult C3HeB/FeJ, C57BL/6N, germ-free (C57BL/6N) and RIP-Cre mT/mG (C57BL/6N) transgenic mice were isolated by collagenase digestion (1 mg/ml #NB8, Serva, Heidelberg, Germany) and rinsed with Hank’s balanced salt solution supplemented with 0.5% BSA. The islets were cultured overnight in RPMI1640 medium (Lonza, Basel, Switzerland) containing 11 mM glucose and supplemented with 10% FCS, 10 mM HEPES, 2 mM l-glutamine, 1 mM Na-pyruvate. Dissociated cell preparations of isolated islets from RipCre mT/mG mice were used to separate green (beta) cells from red (non-beta) cells by fluorescence-activated cell sorting (FACS). FACS was performed with a BD FACS Aria cell sorter (BD Biosciences, Heidelberg, Germany) using BD Diva Software. Cells were sorted with a 100 µm nozzle on a high-purity sort option, and sheath pressure was set to 20 psi. The enrichment of insulin and glucagon/somatostatin mRNA levels was used to confirm efficient separation. The generation of transgenic mice, animal handling, islet isolation and experimentation were approved by the review board of the Land Baden-Württemberg (Regierungspräsidium Tübingen). All animal experiments were performed in compliance with the guidelines and regulations for the welfare of experimental animals issued by the local committee (Notification in accordance with §4 Abs. 3 TierSchG from 21.02.2014 and 19.10.2016 to the Regierungspräsidium Tübingen, Referat 35, Konrad Adenauer Strasse 20, 72072 Tübingen by the Animal Welfare Officer of the University of Tübingen).

### INS-1E cell culture

INS-1E cells, kindly provided by P. Maechler and C.B. Wollheim (University of Geneva, Switzerland) were cultured in RPMI1640 medium (Lonza, Basel, Switzerland) containing 11 mM glucose and supplemented with 10% FCS (Serva, Heidelberg, Germany), 10 mM HEPES, 2 mM l-glutamine, 1 mM Na-pyruvate and 10 µM 2-mercapthoethanol. INS-1E cells and islets were pretreated with 100 ng/ml of pertussis toxin (PTx) for 24 h to block the G_i/o_-dependent pathway. Cells were transfected with siRNA against *FFAR3* (ON-TARGETplus rat FFAR3 (365228), individual; Dharmacon Inc, Chicago, USA) or control siRNA (ON-TARGET plus Non-targeting Pool, Dharmacon) using DharmaFect Transfection Reagent 3 (Dharmacon). Cells were analysed two days after transfection. We used the PCR Mycoplasma Test Kit (AppliChem, Darmstadt, Germany) to ensure that the INS-1E cell line was free of mycoplasma.

### Semi-quantitative analysis of cellular mRNA

For cellular mRNA detection and quantification, islets, pseudoislets, sorted islet cells and INS-1E cells were lysed and the Nucleospin RNA isolation kit (Macherey Nagel, Düren, Germany) was used to isolate RNA. Following an evaluation of RNA integrity (Agilent Technologies, Santa Clara, CA, USA), cDNA of 0.1 µg RNA was synthesised using the Transcriptor first strand cDNA synthesis kit (Roche Diagnostics, Rotkreuz, Switzerland). Semi-quantitative PCR was performed with the LightCycler 480 system (Roche Diagnostics) using the primers (Invitrogen, Carlsbad, CA, USA) listed in Supplementary Table S2.

### Measurement of insulin and glucagon secretion

Isolated islets (5 islets/0.5 ml), pseudoislets (1 pseudoislet/0.1 ml) or INS-1E cells (2 × 10^5^ cells/0.5 ml) were pre-incubated in Krebs–ringer buffer (KRB) containing 2.8 mM glucose as described previously^59^. INS-1E cells and islets were then incubated in the presence of test substances. These were comprised of SCFAs such as acetate (sodium acetate, Millipore, Burlington, MA, USA), propionate (sodium propionate, Sigma-Aldrich, Munich, Germany) and butyrate (sodium butyrate, Millipore, Burlington, MA, USA); the synthetic allosteric FFA3-agonists FHQC (4-(furan-2-yl)-2-methyl-5-oxo-*N*-(*o*-tolyl)-1,4,5,6,7,8-hexahydroquinoline-3-carboxamide)^40^, the orthosteric FFA3-agonist 1-MCPC (1-methylcyclopropane carboxylate)^41^ (Sigma-Aldrich, Schnelldorf, Germany), the allosteric FFA2-agonist 4-CMTB ((*S*)-2-(4-chlorophenyl)-3-methyl-*N*-(thiazol-2-yl)butanamide)^38^, the orthosteric FFA2-agonist TUG-1375 ((2*R*,4*R*)-2-(2-chlorophenyl)-3-(4-(3,5-dimethylisoxazol-4-yl)benzoyl)thiazolidine-4-carboxylic acid)^39^ and the allosteric FFA2-antagonist CATPB ((*S*)-3-(2-(3-chlorophenyl)acetamido)-4-(4-(trifluoromethyl)phenyl)butanoic acid)^36^ (Sigma-Aldrich, Munich, Germany) were synthesized as previously described in the cited literature or purchased from the indicated provider. Properties of synthetic ligands are shown in Table 1. FR900359 was prepared as previously described, used at a final concentration of 1 µM and added to the cell preparations 1 h before the incubation^43^. A radioimmunoassay (Millipore, Burlington, MA, USA) or a sensitive ELISA (Mercodia, Uppsala, Sweden) was used to measure insulin and glucagon in the supernatant and in the islets/cells following extraction with acid ethanol (80%(v/v) ethanol).

### Statistical analysis

Data are presented as mean ± SEM, and the analysis was performed in GraphPad Prism (Graphpad Software, Inc, La Jolla, CA, USA) using ANOVA and Tukey’s as post-test. Student’s unpaired t-test was performed to facilitate a comparison between the two groups. Deviations of p < 0.05 were considered statistically significant.

## Supplementary information

Supplementary Information.
