# Supplementary material for: FFA2-, but not FFA3-agonists inhibit GSIS of human pseudoislets: a comparative study with mouse islets and rat INS-1E cells
Source: Sci Rep. 2020 Oct 5;10:16497. doi: 10.1038/s41598-020-73467-5 (PMC7536384; doi:10.1038/s41598-020-73467-5)
Supplement: Supplementary file 1 — Supplementary Information. [file 41598_2020_73467_MOESM1_ESM.pdf]

FFA2-, but not FFA3-agonists inhibit GSIS of human pseudoislets – a comparative study with mouse islets and rat INS-1E cells.

Estela Lorza-Gil*, Gabriele Kaiser, Elisabeth Rexen Ulven, Gabriele M. König, Felicia Gerst, Morgana Barroso Oquendo, Andreas L. Birkenfeld, Hans-Ulrich Häring, Evi Kostenis, Trond Ulven and Susanne Ullrich

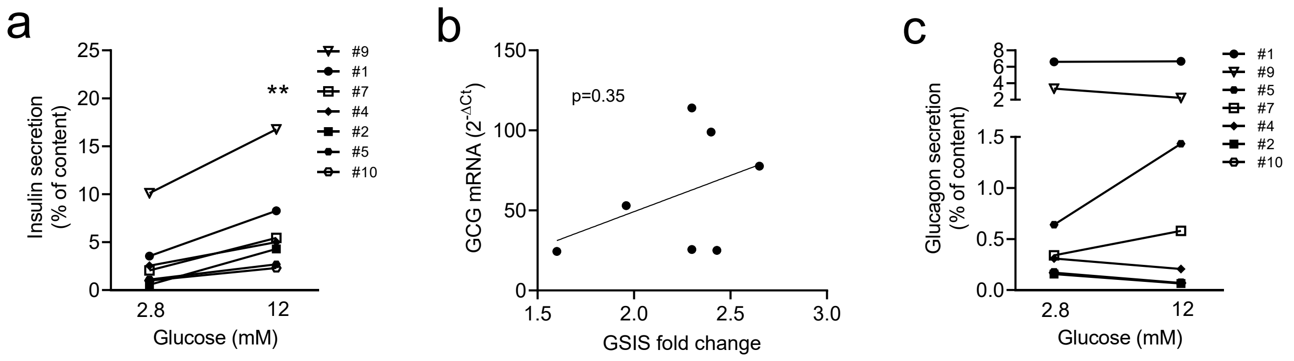

Supplementary Figure S1. Responsiveness of isolated human islets to glucose. Human isolated islets were incubated at low glucose (2.8 mM) and high glucose (12 mM) as described in methods. (a) Glucose stimulated insulin secretion (GSIS) from human pancreatic islets. (b) Correlation between GSIS and GCG mRNA levels. (c) Glucose inhibited glucagon secretion from human pancreatic islets. Significance **p<0.01 unpaired Student's T-test.

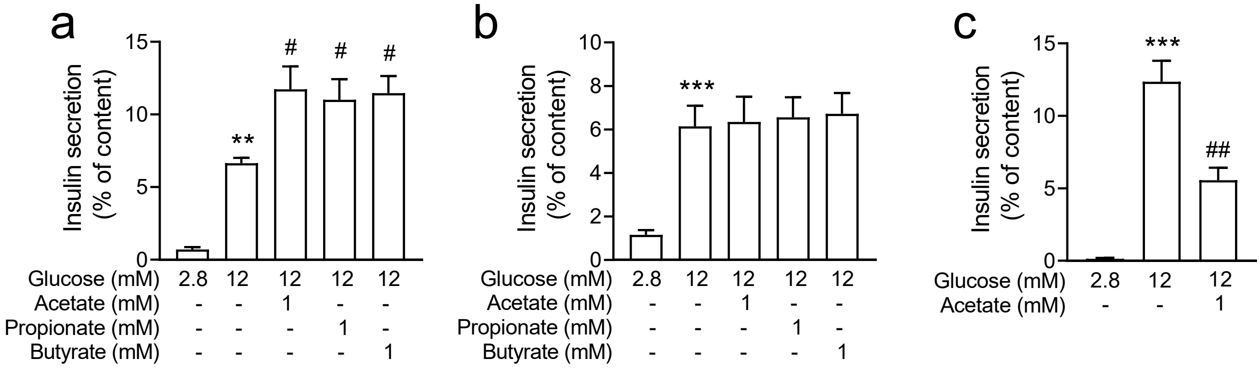

Supplementary Figure S2. Highly variable effects of SCFAs on GSIS in human pseudoislets. Human pseudoislets were prepared and incubated with SCFAs (acetate, propionate or butyrate) for 1 h, as indicated and described under methods. (a) Human pseudoislets from donor #1, donor #2 and donor #3, (b) from donor #4, donor #5, donor #6, donor #7 and donor #11 and (c) from donor #8. Results are presented as mean ± SEM of 4 replicates/conditions/donors. Significance **p<0.01, ***p<0.001 vs 2.8 mM glucose; #p<0.05, ##p<0.01 vs 12 mM glucose, one-way ANOVA, followed by Tukey´s test.

Supplementary Table S1. Characteristics of human pancreatic donors and islet preparations.

Donor	Sex	Age (years)	BMI (kg/m^2)	Islet purity (%)	Islet viability (%)	Study done	Source
#1	M	48	27.7	90	95	Fig. 1a,c,g,h,i; Suppl. Fig. S1,S2	ECIT
#2	F	43	34.1	90	95	Fig. 1a-c,g,i; Suppl. Fig. S1,S2	Tebu-bio
#3	F	48	21.5	90	95	Fig. 1a-c,g,i; Suppl. Fig. S2	Tebu-bio
#4	M	62	24.5	80	95	Fig. 1a-c,g,h,i; Suppl. Fig S1,S2	ECIT
#5	F	53	21.8	85	95	Fig. 1a-c,f,g,i; Suppl. Fig. S1,S2	ECIT
#6	M	55	28.1	85	95	Fig. 1a-c,d,g,i; Suppl. Fig. S2	Tebu-bio
#7	M	58	27.2	80	90	Fig. 1a-c,d,g,h,i; Suppl. Fig S1,S2	ECIT
#8	F	61	24.5	90	95	Fig. 1a-c,e,f,g,i; Suppl. Fig. S2	Tebu-bio
#9	F	63	19.5	90	95	Fig. 1a-c,d,f,h,i; Suppl. Fig. S1	ECIT
#10	F	43	31.5	90	95	Fig. 1a-c,i; Suppl. Fig. S1	Tebu-bio
#11	F	29	26.2	95	95	Fig. 1a-c,d,g,i; Suppl. Fig. S2	Tebu-bio

Supplementary Table S2. Primers and probes used for RT-qPCR

Species	Gene	Roche Probes	Upstream primer	Downstream primer
Human	*FFAR1*	26	5'-TCACCCCTAGCCTGGTCTAC-3'	5'-TTCAGGGGCAGAGAGACTGT-3'
	FFAR2	12	5'-TGTCTCCCCTGCTAAACTGG-3'	5'-GCCTGTGTGGGGTACCAT-3'
	FFAR3	77	5'-TGGGGTCTCAAAGAAGCAGT-3'	5'-GTGATTGCCGGAGAAGTAGG-3'
	FFAR4	13	5'-GCTCATCTGGGGCTATTCG-3'	5'-GCAAATCGAAATTTCCTGGT-3'
	RPS13	68	5'-GGTTGAAGTTGACATCTGACGA-3'	5'-CTTGTGCAACACCATGTGAA-3'
Mouse	*Ffar1*	50	5'-CATCACTCTGCCCCTGAAG-3'	5'-AAGGCAAAGACTGGGCAGA-3'
	Ffar2	9	5'-TTTCCTCGAGCTTGGAAATG-3'	5'-CCATCTTTTCCATTGGCTTC-3'
	Ffar3	12	5'-GTGCACTCACAAGGACTCTCC-3'	5'-AAATTCGGGGTTTATGAGAGG-3'
	Ffar4	45	5'-TTGGTGTTGAGCGTCCTG-3'	5'-CCAGCAGTGAGACGACAAAG-3'
	Ins	32	5'-GAAGTGGAGGACCCACAAGT-3'	5'-AGTGCCAAGGTCTGAAGGTC-3'
	Gcg	27	5'-CCAGTGATGTGAGTTCTTACTTGG-3'	5'-CAATGGCGACTTCTTCTGG-3'
	Sst	53	5'-CCCAGACTCCGTCAGTTTCT-3'	5'-GGGCATCATTCTCTGTCTGG-3'
	Rps13	110	5'-TGCTCCCACCTAATTGGAAA-3'	5'-CTTGTGCACACAACAGCATTT-3'
Rat	*Ffar1*	65	5'-TCATAAACCCGGACTTAGAAGG-3'	5'-TCCAGGCTCCTGTGATGAG-3'
	Ffar2	82	5'-AACGGGAAGCCTCGTTCT-3'	5'-TTCAGGGGTTTCTTCCACCT-3'
	Ffar3	17	5'-CAAGTTCCAAGCCGACTTTC-3'	5'-GTCCAAGGCACACAAGCTCT-3'
	Ffar4	3	5'-TGATCAGCTACTCCAAGATTTACA-3'	5'-GAAGAGCGTTCGGAAGAGC-3'
	Rps13	12	5'-CTGACGACGTGAAGGAACAA-3'	5'-TCACAAAACGGACCTGTGC-3'
